# Comparative transcriptome analysis and identification of candidate R2R3-MYB genes involved in anthraquinone biosynthesis in *Rheum palmatum* L.

**DOI:** 10.1186/s13020-024-00891-4

**Published:** 2024-02-06

**Authors:** Xia Zhao, Feng Yan, Yi-min Li, Jing Tang, Xiao-chen Hu, Zhao Feng, Jing Gao, Liang Peng, Gang Zhang

**Affiliations:** 1 Key Laboratory for Research and Development of “Qin Medicine” of Shaanxi Administration of Traditional Chinese Medicine, Shaanxi University of Chinese Medicine, Xianyang, 712046 China; 2 College of Pharmacy and Shaanxi Qinling Application Development and Engineering Center of Chinese Herbal Medicine, Shaanxi University of Chinese Medicine, Xianyang, 712046 China; 3 State Key Laboratory of Research and Development of Characteristic Qin Medicine Resources (Cultivation), Shaanxi University of Chinese Medicine, Xianyang, 712083 China

**Keywords:** Comparative transcriptomics, Gene family, Anthraquinone biosynthesis, R2R3-MYB, *Rheum palmatum*

## Abstract

**Background:**

*Rheum palmatum* L. has important medicinal value because it contains biologically active anthraquinones. However, the key genes and TFs involved in anthraquinone biosynthesis and regulation in *R. palmatum* remain unclear.

**Methods:**

Based on full length transcriptome data, in this study, we screened the differentially expressed genes in the anthraquinone biosynthesis pathway. The R2R3-MYB family genes of *R. palmatum* were systematically identified based on full-length transcriptome sequencing followed by bioinformatics analyses. The correlation analysis was carried out by using co-expression analysis, protein interaction analysis, and real-time fluorescence quantitative analysis after MeJA treatment. The *RpMYB81* and *RpMYB98* genes were amplified by RT-PCR, and their subcellular localization and self-activation characteristics were analyzed.

**Results:**

Comparative transcriptome analysis results revealed a total of 3525 upregulated and 6043 downregulated DEGs in the CK versus MeJA group; 28 DEGs were involved in the anthraquinone pathway. Eleven *CHS* genes that belonged to the PKS family were differentially expressed and involved in anthraquinone biosynthesis. Twelve differentially expressed MYBs genes were found to be co-expressed and interact with *CHS* genes. Furthermore, 52 *MYB* genes were identified as positive regulators of anthraquinone biosynthesis and were further characterized*.* Three *MYB* genes including *RpMYB81*, *RpMYB98*, and *RpMYB100* responded to MeJA treatment in *R. palmatum*, and the levels of these genes were verified by qRT-PCR*. RpMYB81* was related to anthraquinone biosynthesis. *RpMYB98* had an interaction with genes in the anthraquinone biosynthesis pathway. *RpMYB81* and *RpMYB98* were mainly localized in the nucleus. *RpMYB81* had self-activation activity, while *RpMYB98* had no self-activation activity.

**Conclusion:**

*RpMYB8*1, *RpMYB98*, and *RpMYB100* were significantly induced by MeJA treatment. *RpMYB81* and *RpMYB98* are located in the nucleus, and *RpMYB81* has transcriptional activity, suggesting that it might be involved in the transcriptional regulation of anthraquinone biosynthesis in *R. palmatum*.

**Supplementary Information:**

The online version contains supplementary material available at 10.1186/s13020-024-00891-4.

## Introduction

*Rheum palmatum* L. is a valuable medicinal herb, and its dried roots and rhizomes, as one kind of “Dahuang” in Traditional Chinese Medicine, are widely used to cure diverse diseases, including alleviating fever, blood detoxification, and eliminating jaundice [1]. Phytochemical research has shown that *R. palmatum* contains various constituents, including catechin, gallic acid, and anthraquinones, etc., that exhibit diverse pharmacological activity [2]. Of these active constituents, anthraquinones possess significant anti-inflammatory [3], antiviral [4], and anticancer properties [5]. In plants, that the biosynthesis of anthraquinone matrices mainly involves the shikimic acid and polyketide pathways, and due to the shared intermediates, the MEP (methylerythritol 4-phosphate) and MVA (mevalonic acid) pathways also participate in the biosynthesis of anthraquinones [6, 7]. Therefore, identification of the functional genes that encode key enzymes involved in the specific biosynthetic pathway is a crucial step to increase anthraquinone production in *R. palmatum*.

The key genes and transcription factors (TFs) involved in anthraquinones biosynthesis have been found in a variety of plants containing anthraquinones, for instance, polyketide synthases (PKSs), type III chalcone synthase (*CHS*) [8–10], *CHS-like* [11], and *ICS* [12]. Meanwhile, key genes involved in regulating anthraquinone biosynthesis have been reported in a number of microbes, like cytochrome 450 in *Rhodococcus pyridinivorans* [13], etc.; the identification of these genes would help understand this pathway in plants [10]. For TFs, *bHLH*, *MYB*_related, and *C2H2* were thought to be involved in the anthraquinone biosynthetic pathway [14]. R2R3-MYB transcription factors are known to play an important role in regulating phenylpropanoid metabolism [15]. For example, *OpMYB1* was found to be involved in the biosynthetic pathways of seco-iridoids, monoterpene indole alkaloids, anthraquinone and chlorogenic acid [16]. In *R. officinale*, comparative transcriptome analysis based on different tissues revealed differentially expressed genes related to anthraquinone, catechin and gallic acid biosynthesis [6]. However, in *R. palmatum*, all of the key genes and TFs required for anthraquinone biosynthesis have not been identified [17].

The biosynthesis of many secondary metabolites increases under MeJA induction [18]. The molecular mechanisms underlying MeJA-induced increases in secondary metabolite syntheses have been clarified, such as anthocyanin accumulation in *Arabidopsis thaliana* and apple [19–21], alkaloid accumulation in the medicinal herb *Dendrobium officinale* [22], and terpenoid backbone biosynthesis and steroid biosynthesis in *Cutleaf groundcherry* [23]. However, the molecular mechanism associated with MeJA-induced promotion of anthraquinone biosynthesis in *R. palmatum* remains unknown.

In this study, full length transcriptome was sequenced and assembled to provide a reference genome to determine gene expression levels in root, rhizome, and leaf in the MeJA group. The expression profiles of candidate genes and TFs were further analyzed in seedlings treated with MeJA. The regulation network of *RpMYBs* and *CHS* genes was investigated. Furthermore, the R2R3-MYB TF family members were identified and analyzed based on full-length transcriptome sequencing, and the differential expression of *RpMYBs* under MeJA treatment was determined. The subcellular localization and transcriptional activity of the two MYBs were determined. These results could be a valuable genetic resource to provide comprehensive insights into the molecular mechanisms of active components, such as anthraquinone, and improve the quality control evaluation for this essential medicinal plant.

## Materials and methods

### Materials

Annual plants and mature seeds of *R. palmatum* were collected in August 2021 from Gansu University of Traditional Chinese Medicine and the Hezheng Medicinal Botanical Garden in Longnan, Gansu Province, China; the plants and seeds were identified by Prof. Benxiang Hu of Shaanxi University of Chinese Medicine.

Seeds of uniform size and fullness were sown in black plastic pots (9 cm in diameter and 12.5 cm in height) with 200 g of peat soil, 4 seeds per pot, at 23 ± 2 °C in a greenhouse with a 16 h day/8 h night photoperiod and a light intensity of 9000 Lx. The seeds were originally watered until the water ran out of the pot bottom, and then an additional 50 mL of water was added every 3 days after that. The roots and leaves of three seedlings that grew uniformly and vigorously were chosen, blended in equal parts, and then quickly frozen in liquid nitrogen before delivery.

The *R. palmatum* seedlings were managed with standard nutrient solutions and treated with 200 μmol L^−1^ MeJA in three triplicates. The control group was treated with standard nutrient solutions only, as previously described by Zhao et al., 2022 [24]. Experimental conditions were the same as that described above. At 30 days post germination, seedlings were collected, snapped in liquid nitrogen, and stored at − 80 °C before RNA isolation.

### Full-length cDNA library preparation, sequencing and assembly

Whole plants from *R. palmatum* at the seedling stage were collected 30 days after germination to isolate total RNA. Total RNA was extracted with TRIzol and treated with RNase-free deoxyribonuclease I (New England BioLabs, https://www.neb.com) at 37 °C for 15 min to remove residual DNA according to the manufacturer's instructions. Agilent Bioanalyzer 2100 (Agilent, https://www.agilent.com) was used to quantify and assess the total RNA. Poly(A) RNA was purified using the Poly(A) PuristTM Kit (ThermoFisher Scientific, https://www.thermofisher.com). Then, the SMART cDNA Library Construction Kit (Clontech, now TaKaRa, https://www.takarabio.com) was used to reverse and transcribe 0.5 μg of poly(A) RNA into cDNA. The SMRT sequencing library was constructed using 3.0 μg size-selected cDNA with the PacBio DNA Template Prep Kit 2.0 (Pacific Biosciences, https://www.pacb.com). Four SMRT cells were used to sequence each SMRT library on a PacBio RSII sequencer using P4C2 reagents provided by Gene Denovo Biotechnology Co. (Guangzhou, China) [25].

After sequencing, the data was analyzed on the online Majorbio cloud platform (www.majorbio.com). The raw data was processed by SeqPrep (https://github.com/jstjohn/SeqPrep) and Sickle (https://github.com/najoshi/sickle) to remove adapter reads and low quality reads. The remaining clean data were assembled by Trinity software (https://github.com/trinityrnaseq/trinityrnaseq/wiki), and the integrity and accuracy of the assembly was evaluated by BUSCO (https://busco.ezlab.org/).

### MeJA treatment and determination of anthraquinones levels

The seedlings of 30-day-old *R. palmatum* were treated with 200 μmol L^−1^ MeJA for 0, 2, 4, 6, 12 and 24 h, and this process was repeated three times. Standard aloe-emodin, rhein, emodin, chrysophanol, and physcion were purchased from Shanghai Yuanye Biotechnology Co., Ltd. The dried samples of *R. palmatum* treated with MeJA at different time points were crushed into fine powder (through No.4 sieve) and mixed; then, 1.000 g of the sample was accurately weighed, placed in a conical bottle with a plug, 45.0 mL of methanol was absorbed, and the sample was weighed again. Following ultrasonic treatment for 30 min (power 500 W, frequency 40 kHz), the sample was placed at room temperature, weighed again, and the lost weight was replaced with methanol; then, the sample was centrifuged at 10500 rpm for 12 min, and the supernatant was passed through a 0.22 μm microporous membrane. According to the liquid phase determination method established by a previous study [26], the quantitative analysis of five anthraquinones (aloe-emodin, rhein, emodin, chrysophanol, and emodin monomethyl ether) was carried out, the peak area of each component was determined, and the mass fraction of each component in the sample was calculated.

### RNA isolation and sequencing

Total RNA was extracted from root, rhizome and leaf of *R. palmatum*, and MeJA treatment and control seedings, with three triplicates, were subjected to Trizol reagent (Invitrogen, USA). The quality of total RNA was determined by a Nanodrop 2000 spectrophotometer and an Agilent 2100 Bio-analyzer. The mRNAs were isolated and enriched from total RNAs with Oligo (dT) magnetic beads and fragmented with a fragmentation buffer to construct the RNA pools. Then, the cDNA library was constructed with the Illumina Truseq^™^ RNA sample prep kit (Illumina, USA). Illumina Novaseq 6000 was employed to sequence the libraries with short reads by Gene Denovo Biotechnology Co. (Guangzhou, China).

### Identification and functional annotation of DEGs

The reference genome was constructed using a full-length assembly of *R. palmatum*. The RSEM software was employed to analyze the expression levels of transcripts [27]. Differential expression analysis was performed using the DESeq2 R package (1.10.1) [28] to identify DEGs among three tissues (leaf, root, and rhizome), and the MeJA-stressed and control samples. The genes with an adjusted P-value < obtained by DESeq2 software were considered to be differentially expressed, and the significance of DEGs was determined based on the absolute value of log2 (Group1/Group2) ≥ 1 as the threshold. We use the GOseq R package to perform GO enrichment analysis of DEGs. The KEGG enrichment analysis of DEGs was carried out by KOBAS software [29].

### R2R3-MYB identification

We used the *Arabidopsis* R2R3-MYB protein sequence as the query sequence to search against *R. palmatum* protein sequences using a local basic local alignment search tool (BLAST), considering those with an E-value less than 1 × 10^−10^ as *R. palmatum* R2R3-MYB protein sequences. The *A. thaliana* R2R3-MYB genes downloaded from TAIR (https://www.arabidopsis.org/) were used as query to identify the gene family members in *R. palmatum*. The sequence that was assembled using trinity software with complete ORFs was used in the database to identify R2R3-MYB genes in *R. palmatum*. Our team already published a list of R1-MYB subfamily members, which was renamed from *RpMYB1* to *RpMYB49* [24]. In this study, we named the identified R2R3-MYB gene family members starting from *RpMYB50* to *RpMYB101*.

### R2R3-MYB protein sequence analysis

The primary and secondary structures of the *R. palmatum* R2R3-MYBs were determined using the online tools ProtParam (https://web.expasy.org/protparam/) and SOPMA (https://npsa-prabi.ibcp.fr/cgi-bin/npsaautomat.pl?page=npsasopma). Signal peptides and transmembrane sections were predicted by SignalP-5.0 and TMHMM (https://services.healthtech.dtu.dk/service.php?SignalP-5.0). The WoLF PSORT was used to predict subcellular localization (https://www.genscript.com/wolf-psort.html). By using MEME 4 with the motif parameter set to 20, the conserved motifs were discovered and displayed by TBtools [30]. Weblogo [31] was used to examine protein sequence loci. The neighbor-joining method (bootstrap repetitions = 1000) performed by MEGA7 was used to build the phylogenetic tree of R2R3-MYBs from *R. palmatum* and *A. thaliana* [32].

### Real-time PCR validation

qRT-PCR was performed on a Roche LightCycle 96 PCR System (Roche Applied Science, Mannheim, Germany) using a One Step SYBR PrimeScript PLUS RT-PCR Kit (TaKaRa Bio, Kyoto, Japan). Relative mRNA expression was analyzed with the comparative Ct method (2^−∆∆CT^) and normalized to *β*-Actin as an internal reference gene. All primers were synthesized by Shenggong Bioengineering Co., Ltd. (Shanghai, China), and their sequences are listed in Additional file 1: Tables S1, S2.

### Co-expression and protein interaction analysis

Based on RNA-seq data from the MeJA group, the anthraquinone production pathway-related genes were identified, and the correlations between these genes and *RpMYBs* in *R. palmatum* were assessed using the Pearson correlation method in SPSS software (version 25) (SPSS Inc., Chicago, IL, USA) and visualized using TBtools [30]. Based on the homologous proteins from *A. thaliana*, protein–protein interaction networks of the R2R3-MYB proteins and anthraquinone biosynthesis pathway-related proteins were predicted using STRING.

### Subcellular localization

The target fragments were amplified by using fresh *R. palmatum* cDNA as template and 1305-RpMYBs-F/R as primers. The target gene and pCAMBIA1305-GFP linearized vector were digested by QuickCut restriction enzyme (1305-RpMYB81 using the restriction endonucleases *Xba* I and *BamH* I; 1305-RpMYB98 using the restriction endonucleases *Spe* I and *BamH* I), and the gel was recovered to purify the product. T4 DNA ligase was used to link the vectors. The ligation product was transferred into *E. coli* DH5ɑ competent cells. Three positive colonies with target products were randomly selected. The cloned plasmid was sent to Beijing Aoke for sequencing.

The correct recombinant vector was transferred into *Agrobacterium* EHA105 competent cells (Shanghai Weidi) by the CaCl_2_ method. The plasmids pCAMBIA1305-GFP, pCAMBIA1305-RpMYB81-GFP, and pCAMBIA1305-RpMYB98-GFP were transformed into the inner epidermis of the second to fourth leaves of *N. benthamiana* by Agrobacterium injection. After co-culture at 25 °C for 36 h, the cells were stained with 10 ug/mL 4',6-diamidino-2-phenylindole (DAPI) for 20 min, and then washed three times with pH 7.2 phosphate buffer. The slides were observed by a Olympus FV3000 laser confocal microscope (Olympus Company, Japan).

### Transcriptional activity analysis

The PGBKT7-RpMYBs-F/R primer was used to amplify the target fragments with the fresh cDNA from *R. palmatum* as the template. The target gene and pGBKT7 vector were double digested by QuickCut restriction enzyme (pGBKT7-RpMYB81 using the restriction endonucleases *BamH* I and *Pst* I; pGBKT7-RpMYB98 using the restriction endonucleases *Ecor* I and *BamH* I), and the expression vector was constructed by the method described above. The recombinant plasmids pGBKT7-RpMYB81 and pGBKT7-RpMYB98 were transformed into AH109 yeast competent cells by the heat shock method, and 50 μL of yeast liquid was coated on a SD/Trp medium plate. After 3 days, the positive strains were cultured in a plate containing SD-Trp-His-Ade and SD-Trp-His-Ade-X-*ɑ*-gal in the dark at 30 °C for 3 ~ 4 days, with empty pGBKT7 used as the control.

### Statistical analysis

Experiments were performed according to a completely randomized design. Significant differences (*p < 0.05) between two independent treatments were determined with the Student’s t-test. All data were analyzed with SPSS software (version 25) (SPSS Inc., Chicago, IL, USA).

## Results

### High-quality reads were obtained from the single molecule sequencing-derived transcriptomic analysis

**Fig. 1:**
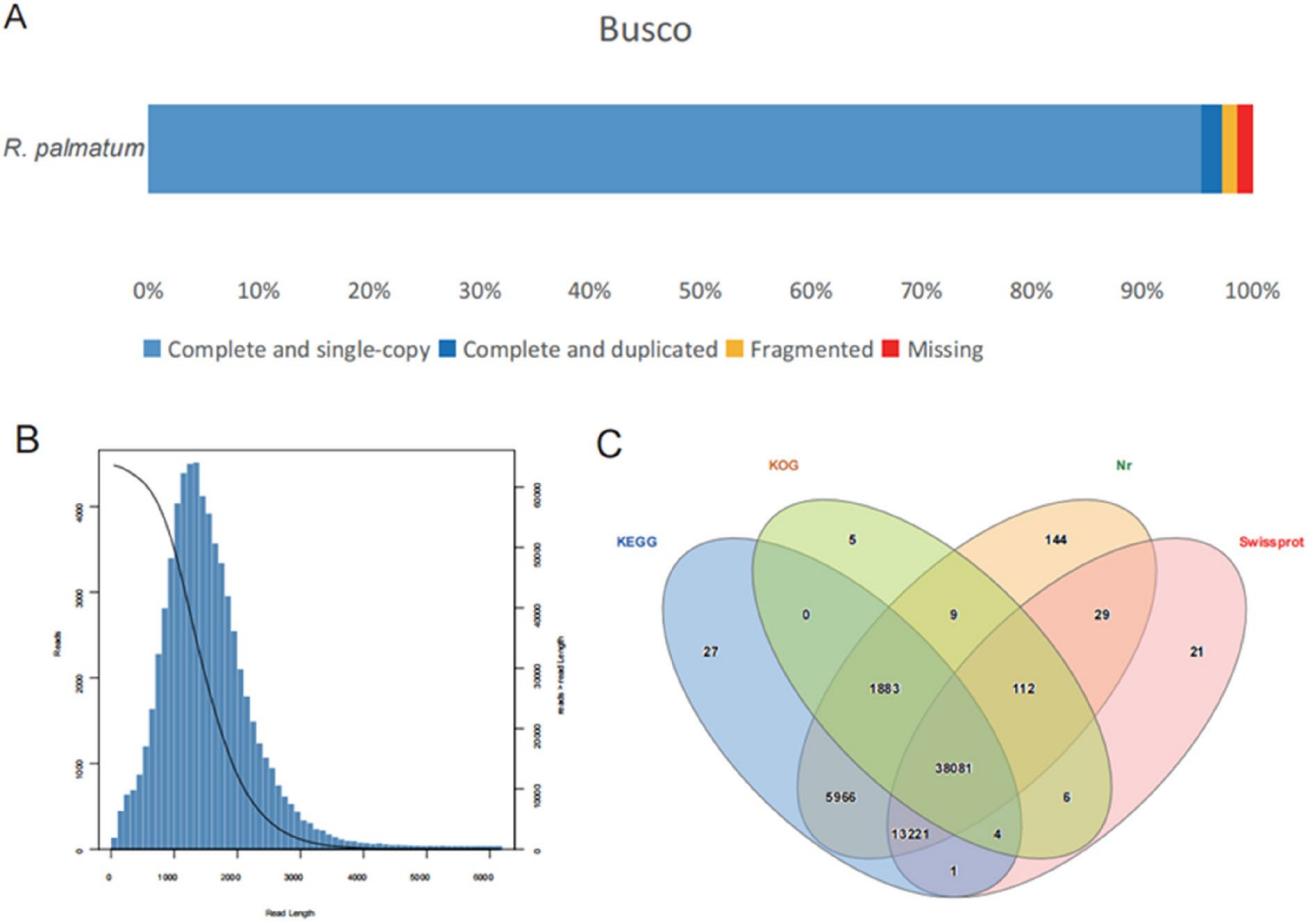
Summary of sequence quality and annotation of the full-length transcriptome of *R. palmatum*. **A** Quality assessment with the BUSCO tool showed proportions classified as complete and single-copy (S, light blue), complete and duplicated (D, blue), fragmented (F, yellow) and missing (M, red). **B** Isoform sequence length distribution. **C** The numbers of protein-coding genes annotated in the NR, UniProt, GO and Pfam databases were illustrated by Venn diagram

To obtain a representative full-length transcriptome for *R. palmatum*, total RNAs extracted from the entire plant were sequenced on four SMRT cells using the PacBio Sequel system. In this study, we generated 1,133,474 reads of insert (ROIs) with a total of 42,912,757 subreads. A total of 77,567 full-length non-chimeric (FLNC) reads within the complete transcript region were acquired. In this study, we obtained 908,028 high-quality CCS reads (> 99% accuracy). Then, we obtained 63,514 isoforms with N50 length 1686 bp (Fig. 1A, B). Quality assessment with the BUSCO tool showed that complete sequences accounted for 84.06% of the conserved core eukaryotic genes (Fig. 1A). In combination with ab initio prediction and homology search, we defined 63,514 unigenes. Of these, a total of 59,509 (93.69%) unigenes were functionally annotated using a suite of functional databases (Fig. 1C). In this study, the relatively high sequence alignment coverage proves that the full-length transcripts obtained by SMRT sequencing was of satisfactory quality.

### The comparative transcriptome analysis among three tissues (leaf, root, and rhizome)

**Fig. 2:**
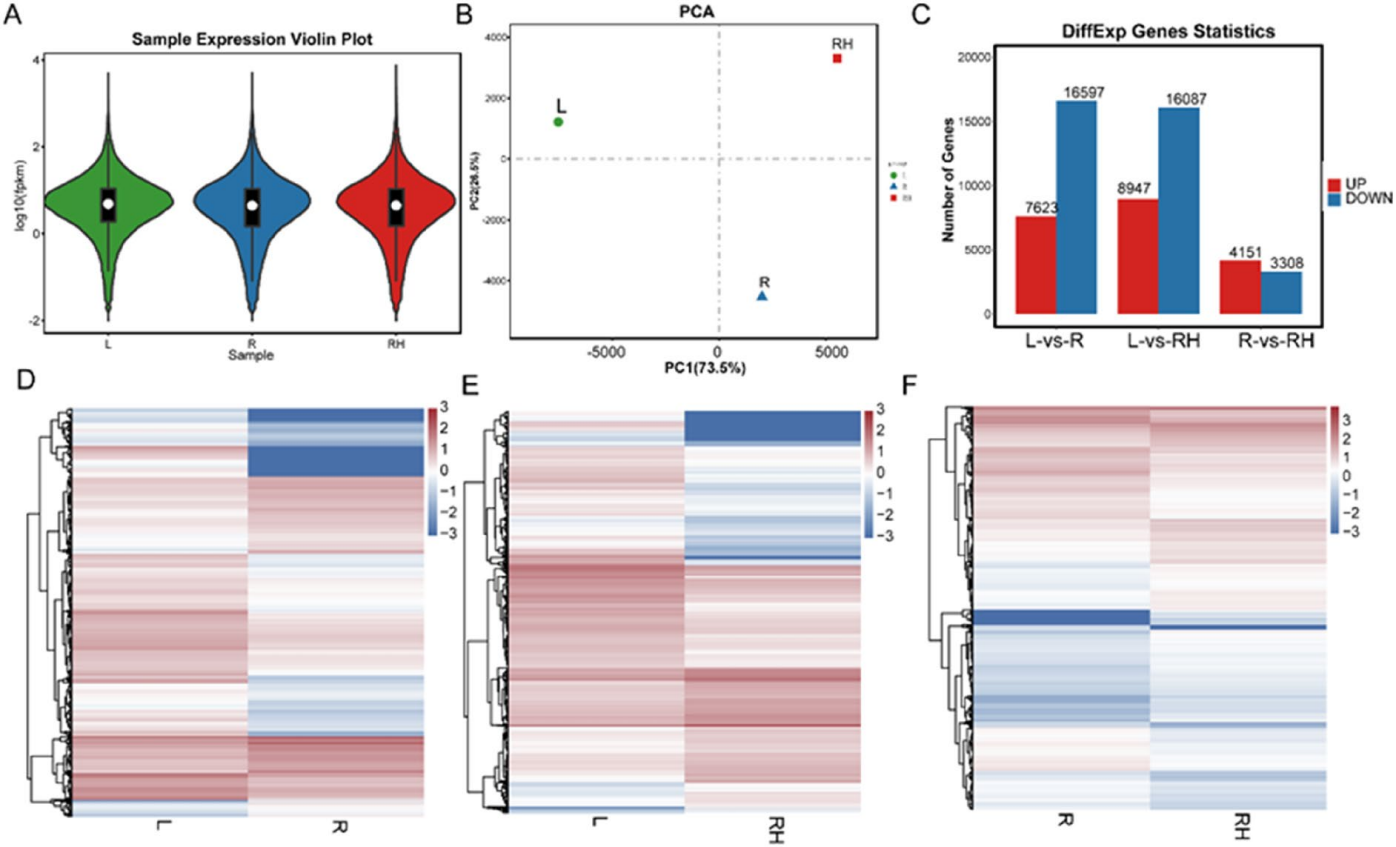
The expression profiling of unigenes expressed in the leaf, root, rhizome of *R. palmatum*. **A** Violin plot of unigenes with mean expression level in leaf, root, and rhizome. **B** The PCA analysis of unigenes based on the expression levels in leaf, root, and rhizome. **C** The DEGs among the three groups of root, rhizome and leaf. **D** The cluster-map of DEGs between leaf and root. **E** The cluster-map of DEGs between leaf and rhizome. **F** The cluster-map of DEGs between root and rhizome. L, leaf; R, root; RH, rhizome

The RNA-seq analysis revealed that the expression patterns of unigenes are similar in leaf, root, and rhizome samples from *R. palmatum* based on mean FPKM value box plots and PCA analysis (Fig. 2A, B). The comparative transcriptomic analysis showed that 7623 upregulated and 16,597 downregulated DEGs in leaves versus root samples, 8947 upregulated and 16,087 downregulated DEGs in leaves versus rhizome samples, and 4151 upregulated and 3308 downregulated DEGs in root versus rhizome samples (Fig. 2C). The cluster-map showed the different expression levels in these three groups, including leaves versus root, leaves versus rhizome, and root versus rhizome (Fig. 2D–F). Results from hierarchical cluster analysis indicated that these DEGs in the stem and leaf samples showed similar expression profiles and clustered into one branch. While in the leaf and root group and the leaf and rhizome group, the DEGs displayed distinct differences in expression levels (Fig. 2D–F).

### Anthraquinone levels in *R. palmtum* seedlings after MeJA treatment

**Fig. 3:**
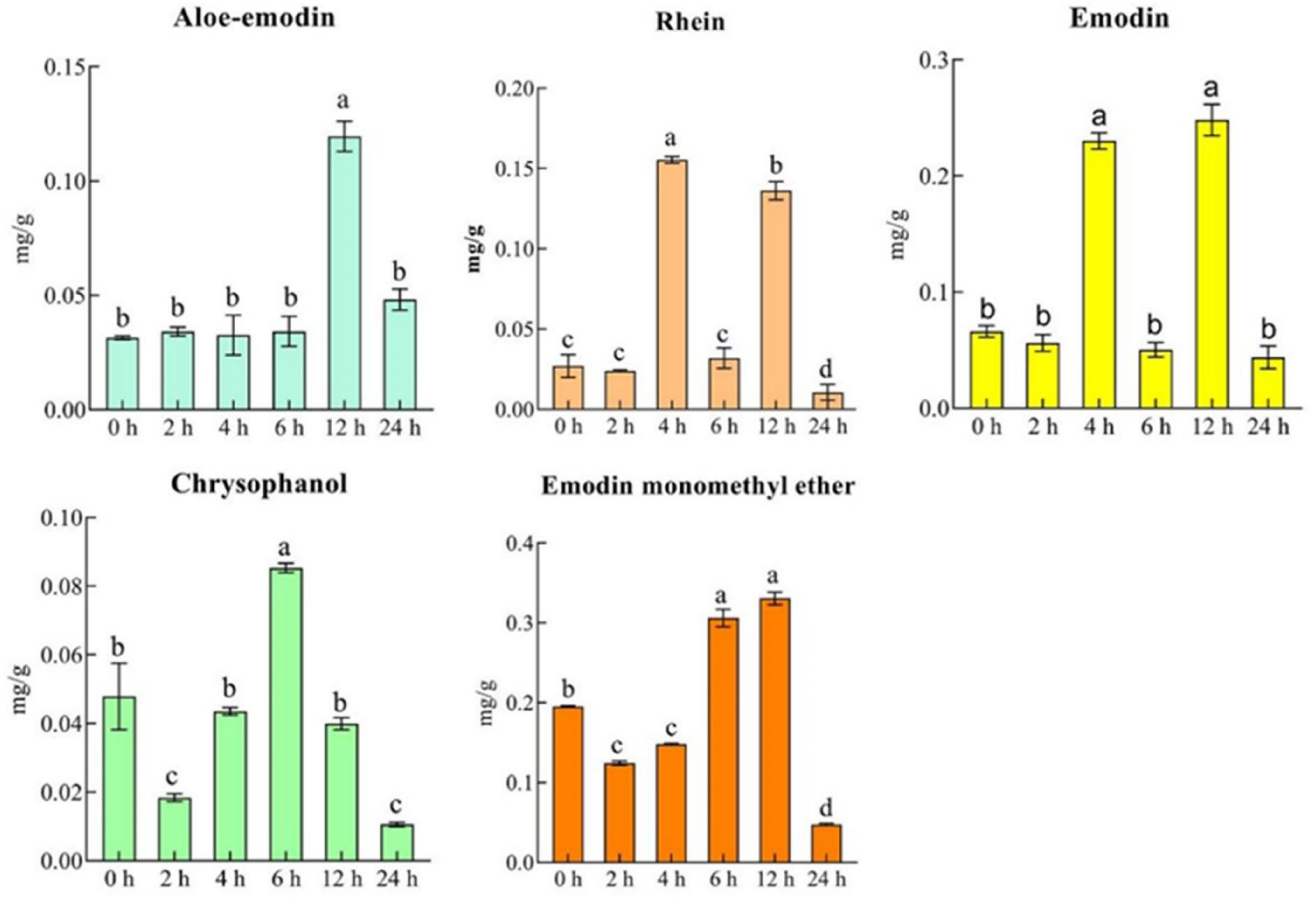
Effects of a MeJA treatment on the levels of five types of anthraquinones (aloe-emodin, rhein, emodin, chrysophanol, and emodin monomethyl ether) in *R. palmatum*

To assess the effect of MeJA treatment on aloe-emodin, rhein, emodin, chrysophanol, and emodin monomethyl ether biosynthesis in *R. palmatum*, the seedlings of 30-day-old *R. palmatum* were treated with 200 μmol L^−1^ MeJA for 0, 2, 4, 6, 12 and 24 h; the contents of the five types of anthraquinones were significantly different (Fig. 3). Except chrysophanol, the levels of the other four components increased significantly at 12 h, among which aloe-emodin, emodin and physcion all reached the highest value, while the levels of rhein and chrysophanol peaked at 4 h and 6 h, respectively (Fig. 3).

### The comparative transcriptome analysis between the CK and MeJA treatment groups of *R. palmatum*

**Fig. 4:**
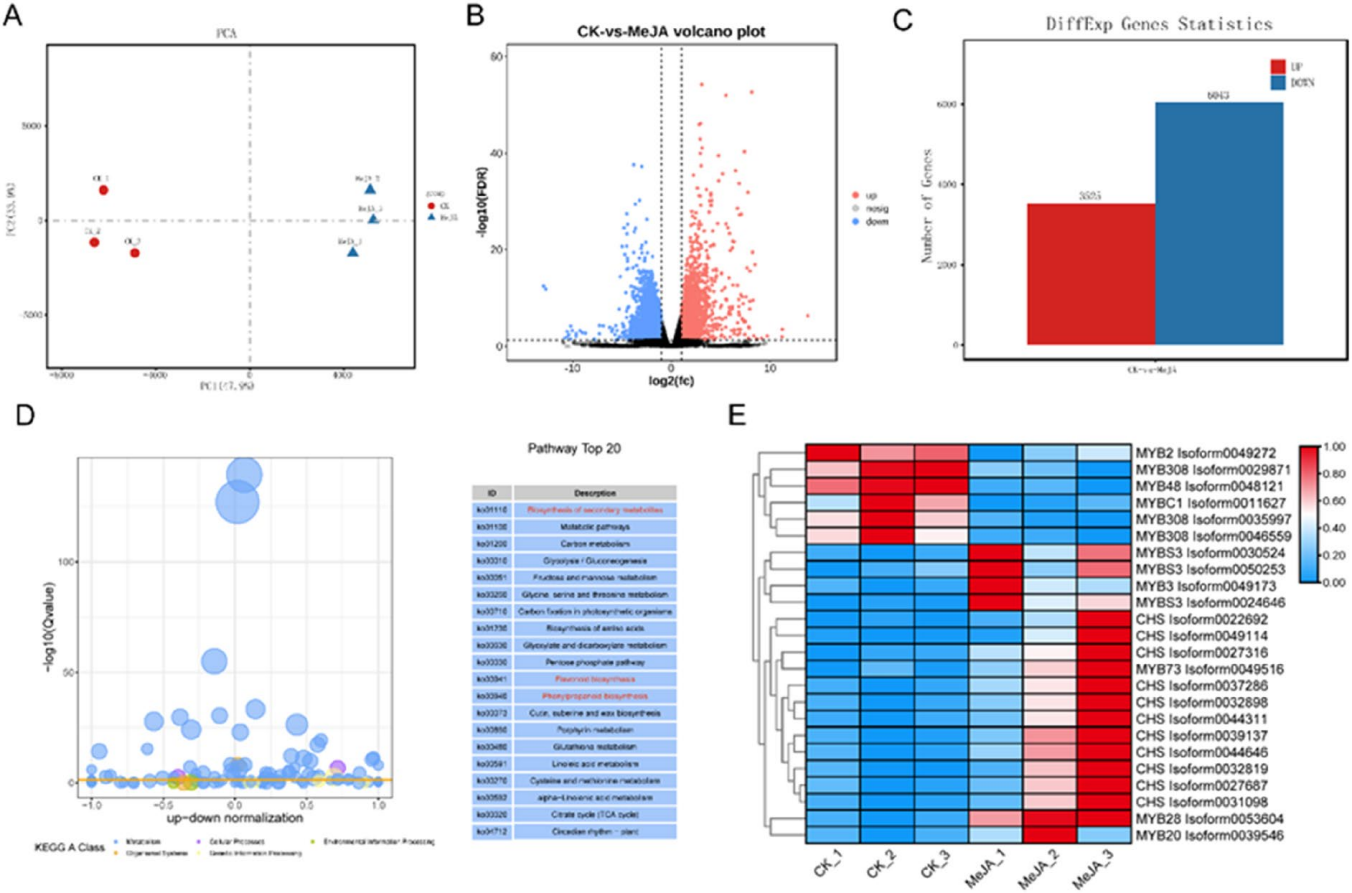
Comparison of the transcriptomes of the CK and MeJA treatment groups of *R. palmatum*. **A** The PCA analysis of unigenes based on the expression levels in the CK and MeJA treatment groups; each treatment was performed in three replicates. **B** The volcano plot of DEGs between the CK and MeJA treatment groups. **C** The statistical analysis of DEGs between the CK and MeJA treatment groups. **D** KEGG enrichment analysis of DEGs between the CK and MeJA treatment groups. **E** The heatmap of differentially abundant MYBs and key enzyme unigenes in the anthraquinone biosynthesis pathway in *R. palmatum*

In the MeJA treatment experiment, PCA analysis based on the average FPKM value showed that there were two separate groups for the CK and MeJA treatment groups (Fig. 4A). Comparative transcriptome analysis showed that there were 3525 upregulated and 6043 downregulated DEGs in the CK group compared with the MeJA treatment group (Fig. 4B, C). KEGG analysis showed enrichment in a wide variety of functional pathways including the biosynthesis of secondary metabolites, flavonoid biosynthesis, and phenylpropanoid biosynthesis, which are related to anthraquinone biosynthesis (Fig. 4D). Of these, 25 DEGs (13 *MYBs* and 11 *CHSs*) related to the anthraquinone biosynthesis pathway showed a different expression level between the CK versus MeJA treatment groups, indicating that the anthraquinone biosynthesis pathway play an important role in the response to MeJA treatment (Fig. 4E). *CHS*, as a type III PKS, plays an important role in anthraquinones biosynthesis via the polyketide pathway. Herein, 11 *CHS* genes and 13 *MYB* genes exhibited differential expression profiles between the MeJA treatment and CK group, indicating that *CHS* and *MYB* are involved in the response to MeJA treatment (Fig. 4E).

### The expression pattern of anthraquinone biosynthesis genes and the gene network

**Fig. 5:**
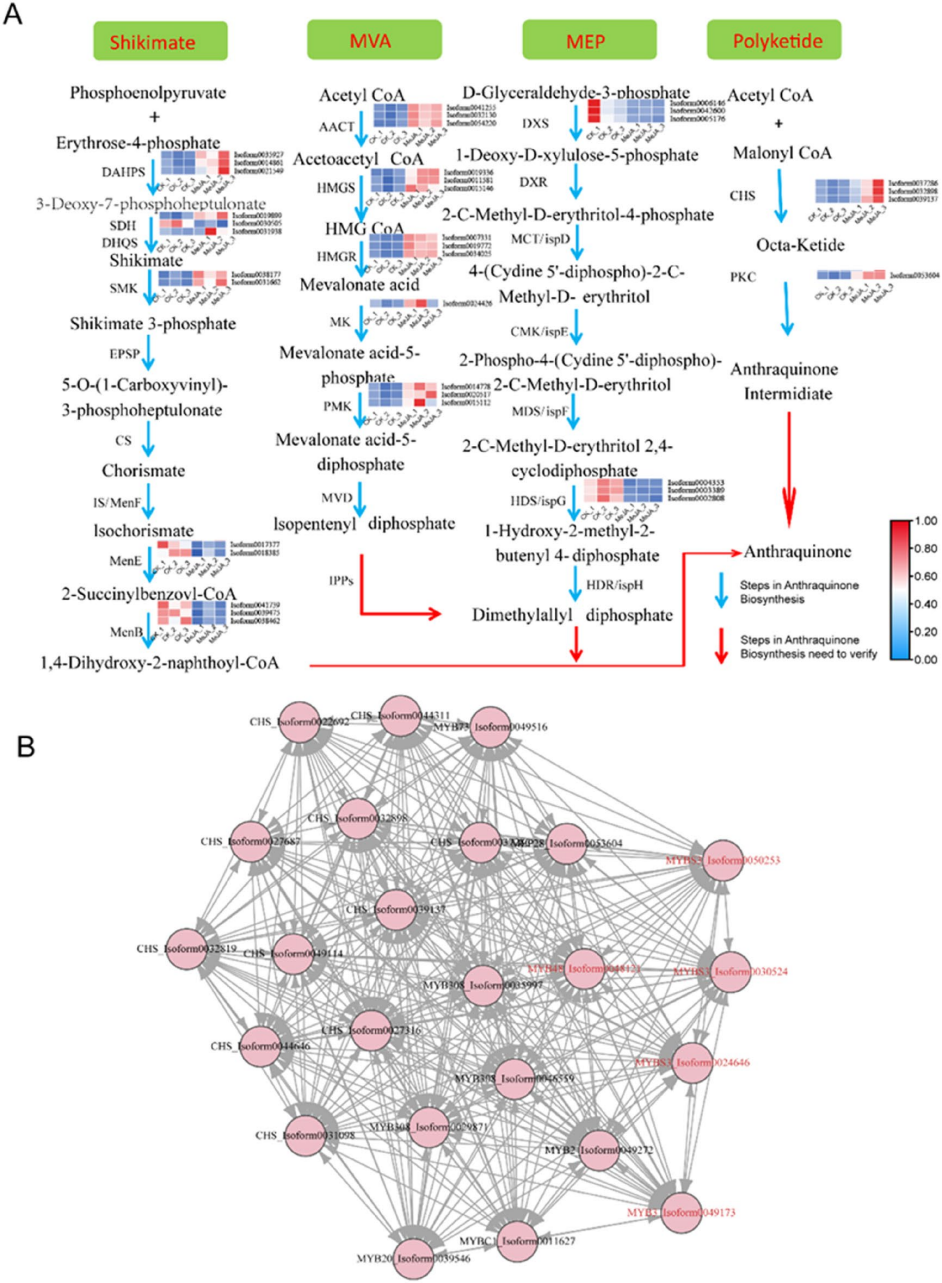
The gene interaction network of DEGs involved in the anthraquinone biosynthesis pathway in *R. palmatum*. **A** The anthraquinone biosynthesis pathway in *R. palmatum*. **B** The gene interaction network of DEGs involved in the anthraquinone biosynthesis pathway

In plants, anthraquinones are synthesized through four pathways, including the shikimic acid pathway, the MVA pathway, the MEP pathway, and the polyketide pathway (Fig. 5A). The differentially expressed genes involved in these four biosynthetic pathways were evaluated following MeJA treatment (Fig. 5A). For MeJA treatment, in the shikimic acid pathway, a total of 5 structural enzymes genes (24 isoforms), including *DAHPS*, *SDH, SMK*, *MenE* and *MenB*, were differentially expressed, either exhibiting upregulated or downregulated expression; in the MVA pathway, the differentially expressed genes, including *AACT*, *HMGS*, *HMGR*, *MK*, and *PMK*, all had upregulated expression, as well as *CHS* and *PKC* genes in the polyketide pathway. Whereas, in the MEP pathway, the only two differentially expressed genes, *DXS* and *HDS/ispG*, had downregulated expression (Fig. 5A).

For the three different tissues, 97 isoforms encoding 11 structural enzymes were identified in the shikimate pathway, and the expression analysis showed that *DAHPS*, *SDH* and *EPSPs* were expressed in the three tissues; *DHQS* was highly expressed in stems and roots; CS was mainly expressed in the roots; and *SMK*, *MenE*, *MenB* and *MenD* were highly expressed in leaves. There were seven enzymes in the MVA pathway. HMGR showed high expression in roots; *AACT* was highly expressed in leaves and stems; *HMGS* and *MVK* were mainly expressed in leaves; and *IPPs* were expressed in all three tissues. We also found that the expression of 81 isoforms encoding seven enzymes in the MEP pathway were similar; all of these isoforms were highly expressed in leaves. In the polyketide pathway, 32 isoforms encoded four enzymes. *CHS* was expressed in all three tissues, the most of which were abundant in leaves, and the rest were much more abundant in roots or rhizomes. *PKS* was highly expressed in roots and rhizomes. Interestingly, one isoform encoding PKS III accumulated the most in leaves. *PKC* was highly expressed in leaves and rhizomes, and two isoforms showed significantly higher expression in rhizomes than in leaves (Additional file 1: Fig S1).

The gene interaction network further showed that 12 *MYBs* interacted with 11 *CHS* genes (Fig. 5B), indicating that *MYBs* might be involved in anthraquinone biosynthesis by regulating *CHS* genes in *R. palmatum* under MeJA stress. *CHS* gene were highly expressed in the three tissues (root, rhizome and leaf) of *R. palmatum*. These results indicate that the *CHS* gene is involved in the physiological process driving the increase in anthraquinones after MeJA treatment, and the *MYB* transcription factor has a strong interaction with *CHS*, indicating that the *MYB* transcription factor may play an important role in the regulation of anthraquinone biosynthesis in *R. palmatum* (Fig. 5). Therefore, investigating the *MYB* family members, gene structure, and gene function involved in the regulation of anthraquinones in *R. palmatum* is needed.

### Characteristics of the *R2R3-MYB* transcription factor and phylogenetic analysis

Based on the full-length transcriptome assembly of *R. palmatum*, we identified a total of 52 full-length R2R3-MYB genes and renamed them as *RpMYB50*–*RpMYB101* (Additional file 1: Table S3). The length of these proteins varied from 237 to 930 amino acids, and their molecular weights ranged from 266.74 kD (*RpMYB101*) to 105.13 kD (*RpMYB50*), with predicted isoelectric points from 5.24 to 9.40 (Additional file 1: Table S3). All R2R3-MYB proteins are likely hydrophilic (GRAVY < 0). Two R2R3-MYB proteins (RpMYB84 and RpMYB87) were stable proteins (instability index < 40), while others were classified as unstable proteins (Additional file 1: Table S3). The aliphatic index ranged from 59.40 to 82.90. The secondary structure analysis revealed that all R2R3-MYBs had *α*-helix, extended chain, *β-*turns and random coiling, and *α*-helix and random coiling were dominant (the mean percentages were 30.23 and 56.63%, respectively), followed by extended chain and *β*-folding (the mean percentages were 8.12% and 5.02%, respectively), which were scattered throughout the proteins (Additional file 1: Table S3). SignalP-5.0 and TMHMM online analyses consistently showed that *R. palmatum* R2R3-MYB proteins were free of signal peptide and transmembrane structural domains (Additional file 1: Table S3). The subcellular localization results showed that *R. palmatum* R2R3-MYB proteins were likely localized in the nucleus (Additional file 1: Table S3).

**Fig. 6:**
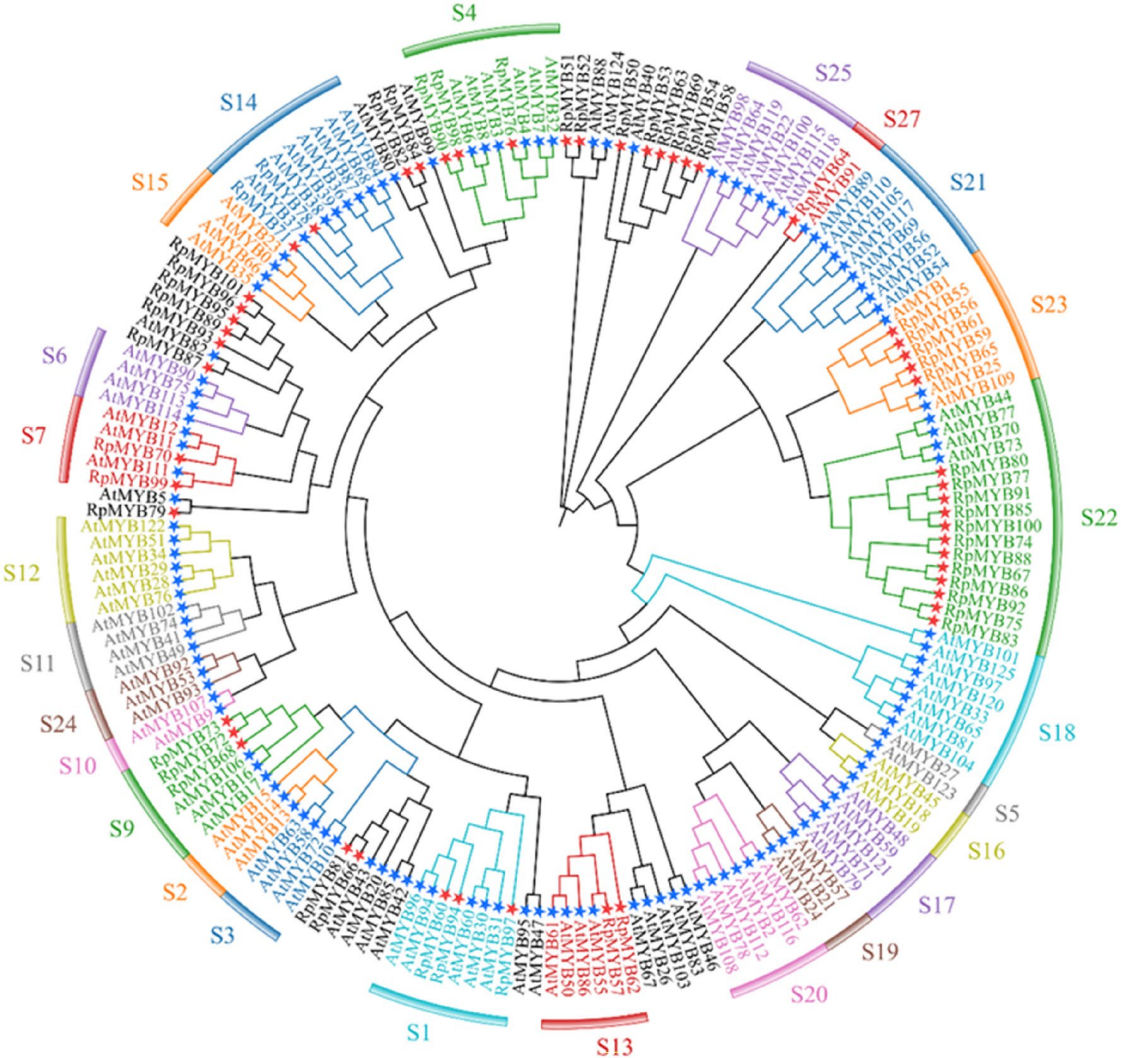
The phylogenetic tree of the R2R3-MYB proteins in *R. palmatum* and *A. thaliana*. The tree was constructed using MEGA 7.0 with the neighbor joining (NJ) method (bootstrap values for 1000 replicates). The colored branches indicate different subgroups. Red stars represent *R. palmatum* R2R3-MYB proteins. Blue stars represent *Arabidopsis* R2R3-MYB proteins. S indicates subfamilies

A phylogenetic tree was constructed based on 52 R2R3-MYBs in *R. palmatum* and 126 AtMYBs, and the results showed that these proteins were divided into 27 subfamilies (Fig. 6). Subfamily 22 contained the most members, with 16 genes. Subfamily 5 contained the fewest members and only had members of *A. thaliana*. The members of subfamily 22 indicated that the genetic relationship between *R. palmatum* and *A. thaliana* is not very close, and they are divided into their own small branches, indicating that there is species differentiation.

### The protein domain and motifs of *R. palmatum* R2R3-MYB proteins

**Fig. 7:**
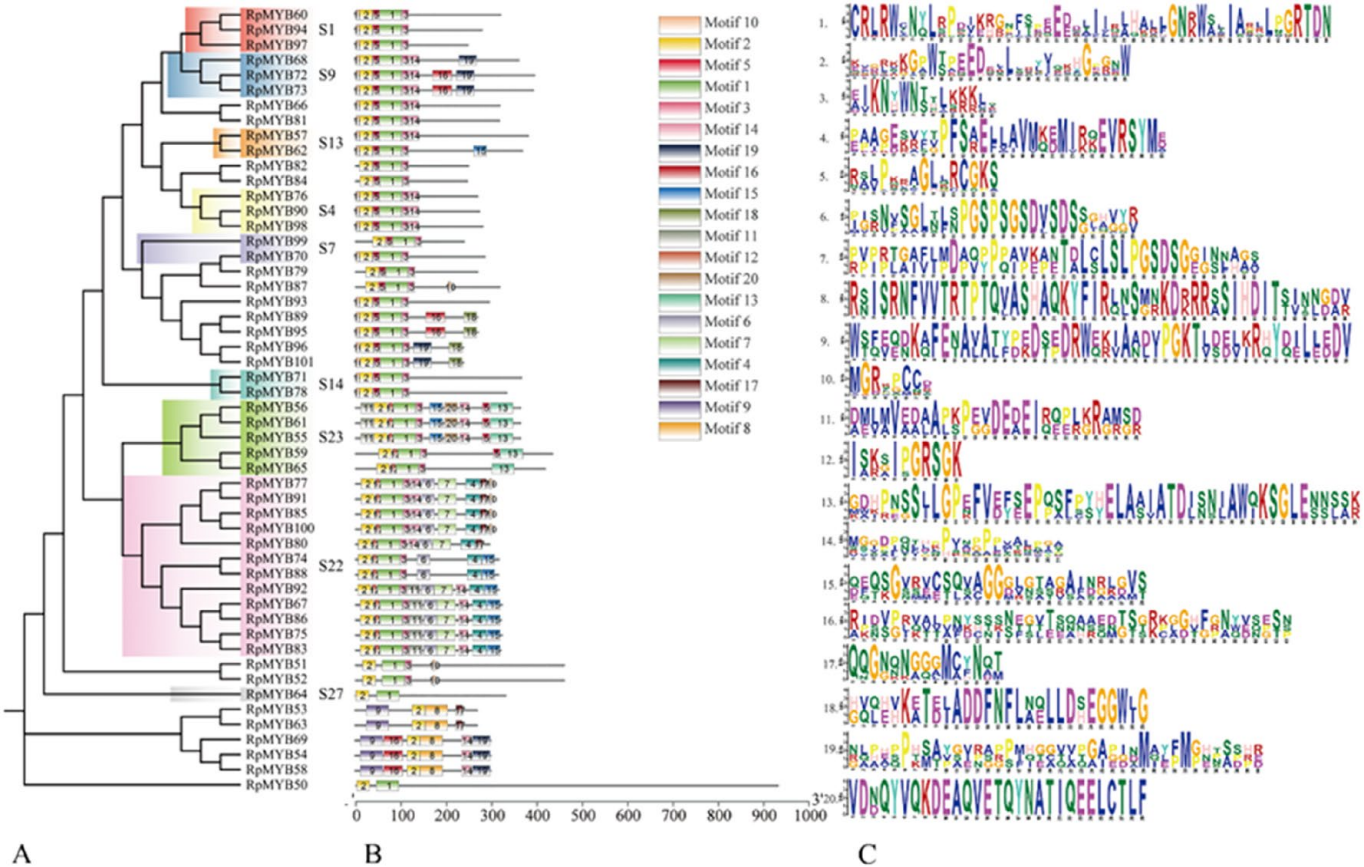
The phylogenetic analysis of R2R3-MYBs in *R. palmatum*. **A** Phylogenetic tree. **B** Conserved motifs. **C** Conserved motif signatures

A total of 52 *R. palmatum* R2R3-MYB proteins were classified into 9 subfamilies, which is consistent with that of *A. thaliana* proteins, namely, S1, S9, S13, S4, 47, S14, S23, S22, and S27 (Fig. 7A, B). The S1, S9, S13, S4, S7, and S14 subfamily R2R3-MYB proteins showed a similar motif composition, which consisted of conserved motif 2, 5, 1, and 3 (Fig. 7A, B). The subfamily 23 proteins were composed of conserved motifs 11, 2, 12, 1, 3, 15, 20, 14, 5, and 13. The subfamily 22 proteins were composed of conserved motifs 2, 12, 1, 3, 6, 7, and another different motif. The subfamily 27 proteins were composed of conserved motifs 2, 12, 1, 3, 6, 7, and another different motif. The motif composition of subfamily 27 proteins varied greatly (Fig. 7A, B). The amino acids of each motif are shown in Fig. 7C.

### Expression profile analysis

**Fig. 8:**
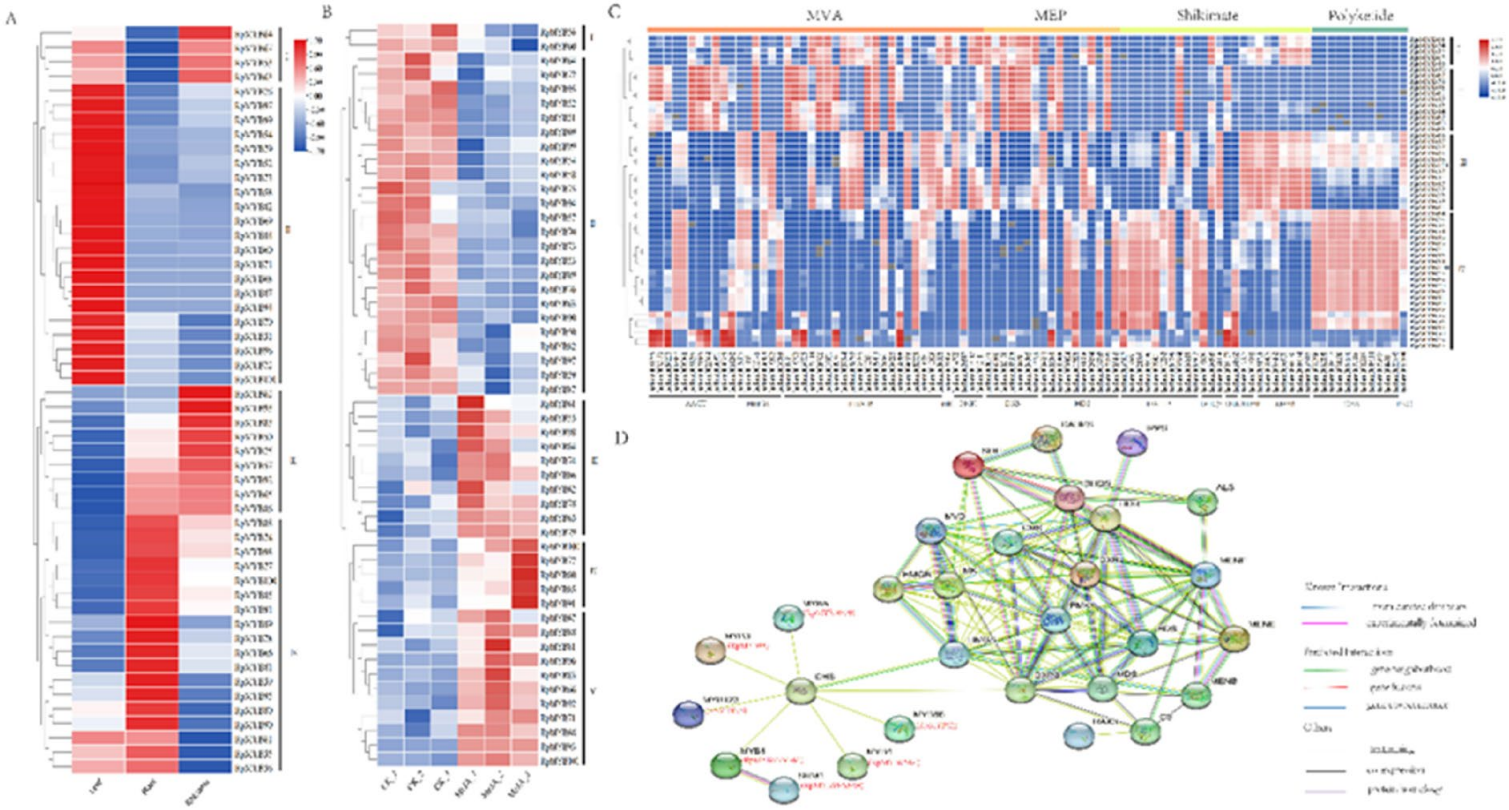
The expression analysis of R2R3-MYBs. **A** The heat map of R2R3-MYBs expression in the different tissues. **B** A heat map of R2R3-MYBs expression in the MeJA group. **C** The Pearson’s correlation coefficients of R2R3-MYBs with proteins in the anthraquinone biosynthesis pathway. **D** The protein interaction network of RpMYBs

The expression profiles of *RpMYBs* in leaves, roots, rhizomes and under MeJA stress were studied (Fig. 8A). The results showed that *RpMYBs* were differentially expressed in different tissues (Fig. 8A). There were 27, 21 and 14 genes that were highly expressed in leaves, roots and rhizomes, respectively (Fig. 8A). According to the expression profiles, the genes can be divided into 4 groups (Fig. 8A). Groups III and IV are mainly highly expressed in roots and rhizomes, indicating that these genes were likely involved in root and rhizome development in *R. palmatum* (Fig. 8A). When *R. palmatum* leaves were treated with MeJA, the *RpMYBs* were divided into five groups according to their expression profiles, of which the genes in groups I and II had downregulated expression, and the genes in groups III, IV and V had upregulated expression (Fig. 8B). The expression of *RpMYB54*, *RpMYB58*, *RpMYB69*, *RpMYB76* and *RpMYB99* was significantly downregulated after MeJA treatment, and the expression of *RpMYB81*, *RpMYB98* and *RpMYB100* was significantly upregulated. Among these genes, *RpMYB81* had the most significantly upregulated expression (Fig. 8B).

The co-expression analysis showed that the correlations between *RpMYBs* and genes related to anthraquinone biosynthesis pathway could be divided into four clusters (Fig. 8C). In cluster I and cluster II, *RpMYBs* showed a strong positive correlation with genes in the MVA and MEP pathways, including *AACT*, *HMGR*, and *DXS*, and negatively correlated with genes in polyketide pathway. *RpMYBs* in cluster I were positively correlated with the downstream genes (*MenE* and *MenB*) of the shikimic acid pathway, while those in cluster II were negatively correlated. *RpMYB98*, which is induced by MeJA and highly expressed in roots, is in this cluster. The gene correlation between *RpMYBs* and the anthraquinone biosynthesis pathway in cluster III was not strong, and the positive correlation mainly involved genes downstream of the shikimic acid pathway. Cluster IV can be divided into 2 subclusters, subcluster 1 had a strong positive correlation with genes in the shikimic acid and polyketide pathways, and subcluster 2 had a strong positive correlation with genes including *AACT, HMGR*, *HDS* and *SMK*. The correlation r values between *RpMYB54*, *RpMYB58*, *RpMYB94*, *RpMYB99*, *RpMYB100* and polyketide pathway genes were all greater than 0.8. These results suggest that *RpMYBs* may have regulate anthraquinone biosynthesis.

Through STRING analysis, CHS, as a hub protein, interacted with some functional MYB proteins, as well as HMGS and DXPS, and these two proteins interacted with 21 enzymes in the anthraquinone biosynthesis pathway. A total of 13 RpMYBs (RpMYB54, RpMYB55, RpMYB56, RpMYB58, RpMYB61, RpMYB62, RpMYB69, RpMYB70, RpMYB79, RpMYB82, RpMYB84, RpMYB93, and RpMYB98) were showed to interact with key enzymes in the anthraquinone pathway, possibly through CHS. Based on its interaction with CHS, RpMYB98 (MYB3) could be implicated in regulating anthraquinone biosynthesis (Fig. 8D). The genes expression profiles in leaf, root, rhizome, and MeJA-treated leaf, and the co-expression network, along with the protein interaction network, demonstrated that two *RpMYBs* (*RpMYB81* and *RpMYB98*) were likely involved in anthraquinone biosynthesis in *R. palmatum* (Fig. 8).

### *RpMYBs* expression analyses under MeJA treatment

**Fig. 9:**
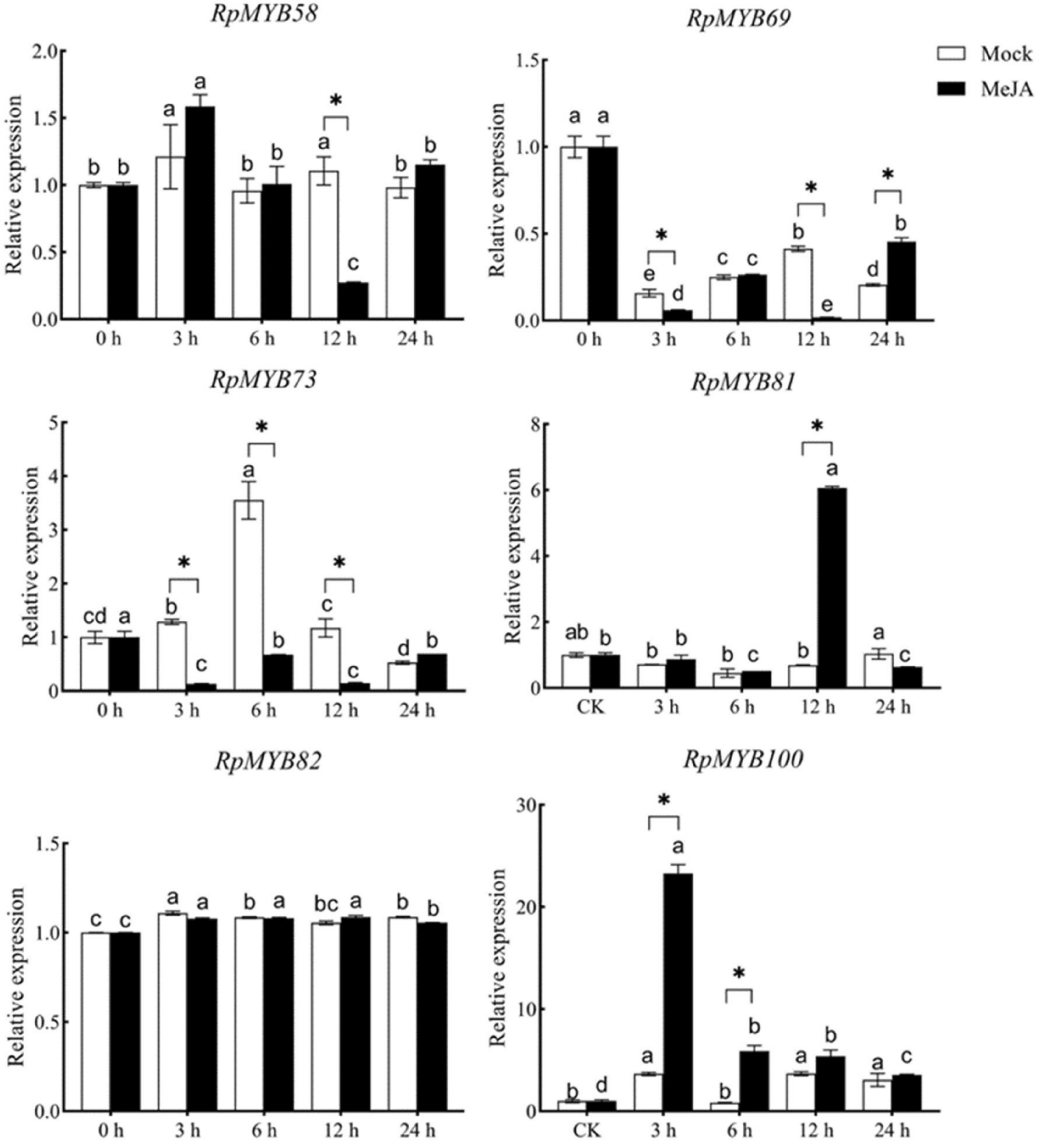
Expression pattern of RpMYB58/69/73/81/82/100 in response to MeJA treatment. MeJA: Methyl jasmonate; The mock groups are the MeJA treatment mock groups. Different lowercase letters (a, b, c, d) and the symbol (*) above the columns represent significant (P < 0.05) differences among MeJA treatments

Six *RpMYBs* were chosen for to validate the RNA-seq analysis result. qRT-PCR analyses were used to determined their expression profiles in the CK and MeJA groups. The results showed that after MeJA treatment, the expression of *RpMYB58* was significantly downregulated at 12 h. *RpMYB69* expression was significantly downregulated at 3 h and 12 h after MeJA treatment, but significantly upregulated after 24 h. The expression of *RpMYB73* were significantly downregulated at 3 h and 12 h. *RpMYB81* expression was significantly upregulated at 12 h. The expression of the *RpMYB100* gene was significantly upregulated at 3 h after MeJA treatment (Fig. 9). Overall, the qRT-PCR expression results were mostly in accordance with those of RNA-seq analyses, indicating that *RpMYB81* was likely involved in anthraquinone biosynthesis in *R. palmatum* (Fig. 9).

### Subcellular localization and transcription activation of *RpMYB81* and RpMYB98

**Fig. 10:**
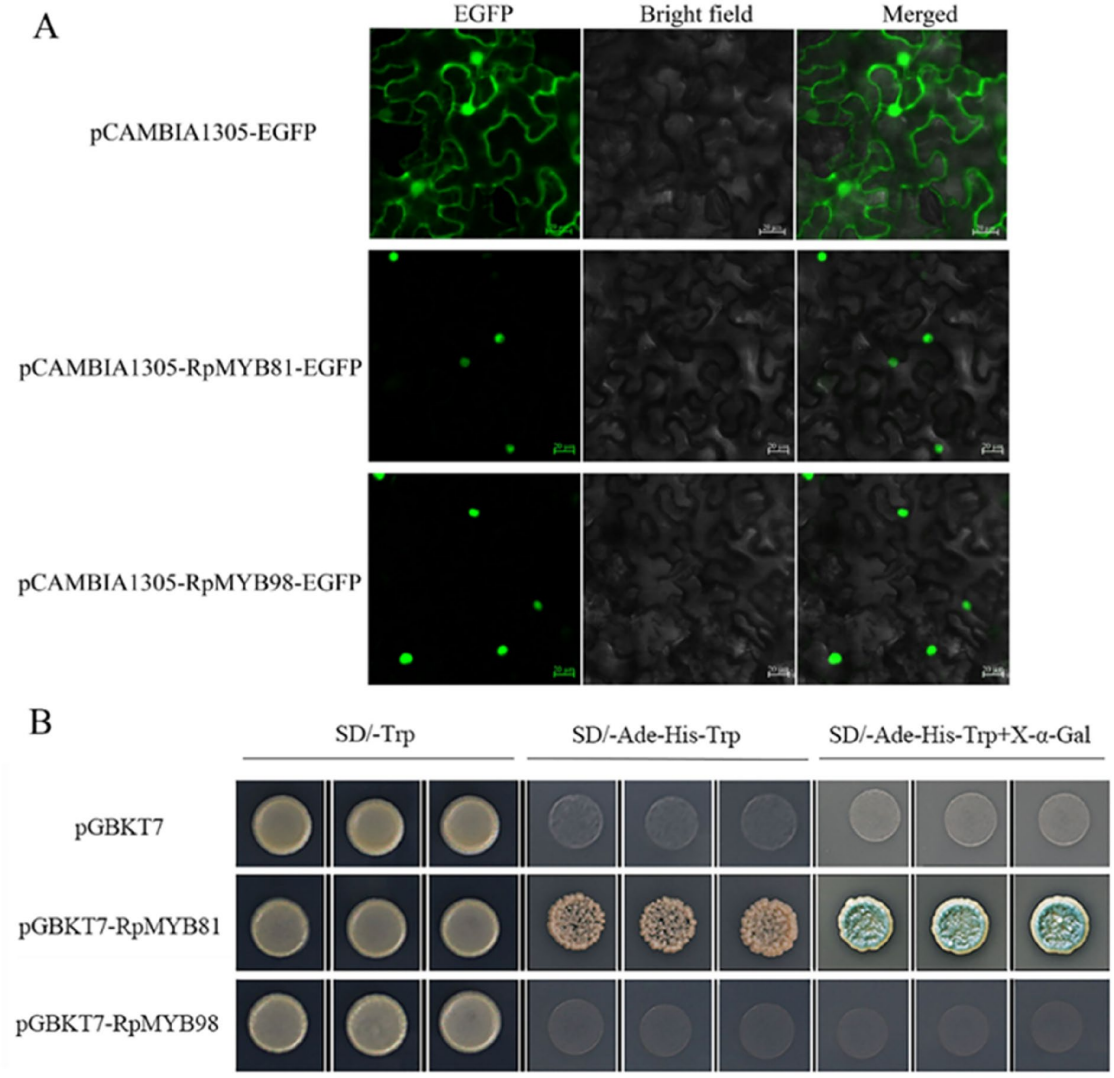
The subcellular localization and transcription activation analysis of RpMYB81 and RpMYB98. **A** Subcellular location of the RpMYB81 and RpMYB98 proteins in *N. benthamiana* lower epidermal cells. The images were taken from three fields of view, including enhanced green fluorescent protein (EGFP), bright light, bright light merged EGFP. Bar = 20 µm. **B** Transcriptional self-activating activity of the RpMYB81 and RpMYB98 proteins in yeast cells. SD/-Trp: synthetic dropout medium without tryptophan; SD/-Ade-His-Trp: synthetic dropout medium without adenine, histidine, tryptophan; and SD/-Ade-His-Trp + X-*α*-Gal: synthetic dropout medium without adenine, histidine, tryptophan but adding 5-bromo-4-chloro-3-indoxyl-*α*-D-Galactopyranoside

Previous bioinformatics results showed that *RpMYB81* and *RpMYB98* were located in the nucleus. To further confirm their subcellular localization, the pCAMBIA1305-RpMYB81-GFP construct, the pCAMBIA1305-RpMYB98-GFP construct and GFP control were separately transfected into epidermal cells of tobacco leaves. Fluorescence microscopy showed that the green fluorescence signal of the GFP control was ubiquitously distributed throughout the cell, whereas the pCAMBIA1305-RpMYB81-GFP and pCAMBIA1305-RpMYB98-GFP fusion proteins were only observed in the nucleus. These results imply that the RpMYB81 and RpMYB98 proteins localized to the nucleus of plant cells (Fig. 10A).

To determine whether RpMYB81 and RpMYB98 had transcriptional activation activity, ORF cDNA was cloned into the pGBKT7 vector for genetic transformation of the yeast strain AH109. The pGBKT7-Rp MYB81 transformant not only grew well on SD/-Trp medium but also grew well on SD/-Trp-His-Ade medium, and showed strong *β*-galactosidase activity in the presence of X-*α*-gal. In contrast, yeast cells containing pGBKT7-RpMYB98 or pGBKT7 grew only on SD/-Trp medium but not on SD/-Trp-His-Ade medium. These results suggest that *RpMYB81* is a transcriptional activator and can activate the expression of downstream genes, while *RpMYB98* does not have self-activating activity (Fig. 10B).

## Discussion

*R. palmatum* can be used as a medicinal plant because it contains virous bioactive compounds such as anthraquinone, anthrone, and flavonoids [2, 33]. In recent years, the research on *R. palmatum* has mainly focused on the extraction of chemical components, traditional efficacy and modern pharmacological effects [34, 35]. Due to the limitation of genomic information, the biosynthetic process of pharmacologically active anthraquinones in *R. palmatum* is still unclear [8]. In this study, we first assemble a high-quality full length transcriptome sequence, which provided a reference sequence for subsequent RNA-seq analysis (Fig. 1). The analysis of potential candidate genes by comparing the whole transcriptome will help to better understand the secondary metabolic pathways [6, 13, 14]. Some functional genes involved in the biosynthesis of secondary metabolites have been elucidated, such as *CYP450*, *bHLH*, and *MYB* [36, 37], as well as *CHS*, *CHS*-like, *CYP450*, and *BGL* in anthraquinone biosynthesis [8, 11]. In our study, we first confirmed that after MeJA treatment for different time periods, the levels of five anthraquinones (aloe-emodin, rhein, emodin, chrysophanol, and physcion) were significantly different. These results confirmed that MeJA was an important factor that altered the levels of anthraquinones (Fig. 2).

To understand the regulatory transcription factors that play an important role in the biosynthesis of anthraquinone in *R. palmatum*, we performed RNA sequencing of the leaf, root, rhizome, and seedlings in CK and under MeJA stress. The DEGs in the leaf, root, and rhizome showed differential expression patterns in a tissue specific manner, which provided a basis to study the molecular mechanism of tissue-specific distribution of secondary metabolites (Fig. 2). A total of 3525 upregulated and 6043 downregulated DEGs between the CK and MeJA treatment groups were further identified as candidate genes; interestingly, some of these genes were related to anthraquinone biosynthesis. In the RNA-seq analysis, genes in the anthraquinone biosynthesis pathway showed different expression patterns, suggesting that the four compound anthraquinone biosynthesis pathways might function differently in a MeJA dependent manner (Fig. 5). In the polyketide pathway, *CHS* genes were induced in response to MeJA treatment; therefore, these genes were involved in anthraquinones biosynthesis in *R. palmatum* following MeJA treatment. Evidence has shown that *CHS* genes are responsible for anthraquinone biosynthesis in plants [9, 10]. Twenty-five DEGs (13 *MYBs* and 11 *CHSs*) were significantly differentially expressed, and further interaction network analysis showed that *MYB* could interact with the *CHS* gene, indicating that the *MYB* transcription factor may play an important role in the regulation of anthraquinones in *R. palmatum* (Fig. 5). Therefore, investigating the *MYB* family members, gene structure, and gene function in regulating anthraquinone biosynthesis in *R. palmatum* in necessary (Fig. 5).

To identify the key candidate *MYB* TFs that regulate anthraquinone biosynthesis, full-length transcriptomic analysis was performed; this analysis provides a promising method for studying non-model plants [38]. Previously, R1-MYB subfamily members were characterized and renamed *RpMYB1* to *RpMYB49* [23]. In this study, 52 R2R3-MYB genes were identified using full-length transcriptome sequencing (Fig. 6). The number of R2R3-MYB genes was lower than that of *Arabidopsis* (126) [39], rice (99) [40], impatiens (73) [41], *Chrysanthemum nankingense* (183) [42], and *Casuarina equisetifolia* (107) [43], but it was equivalent to the reported number in *Morinda officinalis* (51) [44] and *Bothriochloa ischaemum* (43) [45] (Fig. 7). Because genes clustered in the same branch have close genetic relationships and similar functions, in this study, we predicted the function of RpMYBs based on the phylogenetic tree. *RpMYB76* was highly homologous to *AtMYB4* in the S4 subgroup, which is involved in inhibiting the synthesis of lignin and flavonoids in the anthraquinone metabolic pathway [46, 47] (Fig. 6). This analysis showed that *RpMYB76* might be involved in phenylpropanoid metabolism and the phenylpropanoid-related anthraquinones metabolic pathway, similar to *OpMYB1* [16] (Fig. 7). *RpMYB70* and *RpMYB99* were homologous to members of S7 (*AtMYB11*, *AtMYB12*, and *AtMYB111*), and these three *Arabidopsis* genes are involved in modulating flavonoid biosynthesis in favor of flavonol accumulation [48]. *AtMYB85* promotes the synthesis of cellulose and lignin in *A. thaliana*, and *AtMYB68* negatively regulates the deposition of lignin in *A. thaliana* roots [39]. *RpMYB66*, *RpMYB71*, *RpMYB78*, and *RpMYB81* were located in the same subfamily with the above transcription factors that regulate lignin synthesis in *A. thaliana*. Thus, these transcription factors in *R. palmatum* likely have similar functions.

The transcriptional expression of most *R2R3-MYB* genes was generally tissue-specific. According to the results of cluster analysis, *RpMYBs* genes were divided into 4 clusters. Cluster 1 contained four *R2R3-MYB* genes, all of which were highly expressed in roots, and cluster 2 genes were highly expressed in roots and rhizomes. Most of the genes in cluster 3 and cluster 4 were highly expressed in leaves and some in rhizomes, suggesting that these genes may not be involved in root development. We further found that *RpMYB81* and *RpMYB98*, which belonged to cluster 4, were highly expressed in roots, indicating that these two genes may play an important role in anthraquinone biosynthesis in roots (Fig. 8).

MeJA is a kind of plant specific signal regulator that is involved in the regulation of growth, development and secondary metabolite synthesis in various plants [18]. The *NtMYBJS1* gene is involved in the growth and development of tobacco via polyamines in a MeJA-dependent manner [49]. In ginseng, after MeJA induction, the accumulation of ginsenosides increased with the upregulation of the expression of key enzyme genes such as *PgDDS* [50]. *SmMYB9b* transcription was activated by MeJA, which greatly increased tanshinone content and lipophilic activity in *salvia capillaris* roots [51]. In this study, *RpMYBs* showed different response patterns under MeJA treatment. *RpMYB81*, *RpMYB98*, and *RpMYB100* were significantly induced by MeJA, suggesting that the three genes may be positive regulators of secondary metabolism pathways (Fig. 6B). A protein interaction network was established between 52 RpMYBs and anthraquinone biosynthesis pathway related enzymes (Fig. 8). The differentially expressed *RpMYB98* (*MYB*3) had significantly upregulated expression after MeJA induction and was clustered with key enzyme genes in the anthraquinone synthesis pathway. *MYB3* is a MYB transcription factor that inhibits the expression of phenylpropanoid biosynthesis genes [52]. Studies have shown that effective inhibition of phenylalanine ammonia lyase activity in the shikimate pathway can change the metabolic flow, activate the synthesis of anthraquinones, and lead to the accumulation of anthraquinones [53]. Interestingly, RpMYB98 is also involved in regulating the expression of key enzyme genes in the MEP pathway. The 1-deoxy-D-xylulose 5-phosphate synthase gene (*DXS*) is one of the rate-limiting enzymes in the MEP pathway. Co-expression analysis showed that RpMYB98 was significantly positively correlated with DXS. SmMYB36/9b in *S. miltiorrhiza* may affect the accumulation of diterpenoid quinone tanshinones by regulating key enzymes in the mevalonate (MVA) pathway and the 2-C-methyl-D-erythritol-4-phosphate (MEP) pathway [54]. Functional genes were further validated and exhibited similar expression patterns in both qRT-PCR and transcriptomic analysis, indicating the reliability of transcriptome data. The *RpMYB81* gene had significantly upregulated expression 12 h after MeJA treatment, as was verified with qRT-PCR (Fig. 9).

Transcription factors have the ability to regulate diverse processes, such as secondary metabolism, by interacting with DNA motifs in the promoter area of genes of interest [37, 55]. This regulation is mediated by TFs interaction with itself or with other proteins, depending on its self-transcriptional activity; this activity is analyzed by yeast one-hybrid assays [55]. In this study, pGBKT7-RpMYB81 and pGBKT7-RpMYB98 were transformed into AH109 to verify whether there was self-activation. The results showed that *RpMYB81* had obvious transcriptional activity, suggesting that it may be involved in the transcriptional regulation of *R. palmatum* or play an independent role in transcriptional activation. *RpMYB98* does not have self-activating activity and can be used for subsequent yeast two-hybrid experiments. This different may be due to containing the specifical inhibitory domain of a TF [56], which requires the participation of auxiliary TFs to form a transcription complex (Fig. 10). Overall, these results identified candidate *MYB* genes which might be involved in transcriptional regulation in *R. palmatum*.

## Conclusion

In the present study, we performed full -length transcriptome sequencing of *R. palmatum* using SMRT sequencing combined with NGS sequencing. DEGs induced by MeJA treatment were identified based on NGS transcriptomics, and the DEGs were functionally enriched in the anthraquinone synthesis pathway. MeJA treatment increased the levels of five types of anthraquinones (aloe-emodin, rhein, emodin, chrysophanol, and emodin monomethyl ether) and upregulated the expression of genes related to the anthraquinone biosynthesis pathways, including *CHS* and *MYB*. In order to identify candidate *R2R3-MYBs* that could regulate the anthraquinone biosynthesis pathway, a total of 52 *R2R3-MYB* were identified based on full-length transcriptomics data. The *RpMYB81* gene had significantly upregulated expression 12 h after MeJA treatment, and this result was validated by qRT-PCR. *RpMYB81* and *RpMYB98* are both located in the nucleus, and RpMYB81 had obvious transcriptional activity, suggesting that it may be involved in transcriptional regulation in *R. palmatum*.

### Supplementary Information

**Additional file 1: Table S1.** Primers used in the qPCR. **Table S2.** Primers used in the gene cloning. **Table S3.** The R2R3-MYB transcription factor characteristics. **Figure S1.** Expression patterns of anthraquinone biosynthesis pathway in different tissues. A: shikimic acid pathway; B; MEP pathway; C: MVA pathway; D: Polyketide pathway.

## Data Availability

The sequencing data have been deposited in the Sequence Read Archive (SRA) under the project PRJNA981643.

## Abbreviations

TF: Transcription factor
ROIs: Reads of insert
FLNC: Full-length non-chimeric
NGS: Next generation sequencing
MEP: Methylerythritol 4-phosphate
MVA: Mevalonic acid
MeJA: Methyl jasmonate
OSB: O-succinylbenzoate
AACT: Acetyl-CoA acetyltransferase
HMGS: 3-Hydroxy-3-methylglutaryl-CoA synthase
HMGR: 3-Hydroxy-3-methylglutaryl-CoA reductase
MK: Mevalonate kinase
PMK: Phosphomevalonate kinase
DXS: 1-Deoxy-D-xylulose-5-phosphate synthase
HDS: 4-Hydroxy-3-methylbut-2-en-1-yl diphosphate synthase
DAHPS: 3-Deoxy-D-arabino-heptulosonate 7-phosphate synthase
DHQS: 3-Dehydroquinate synthase
SMK: Shikimate kinase
MenE: 2-Succinylbenzoate–CoA ligase, chloroplastic
MenB: Naphthoate synthase
CHS: Chalcone synthase
PKC: Polyketide cyclase
PKS: Type III polyketide synthase
BGL: *β*-Glucosidase
