# Supplementary material for: Comparative transcriptome analysis and identification of candidate R2R3-MYB genes involved in anthraquinone biosynthesis in Rheum palmatum L
Source: Chin Med. 2024 Feb 6;19:23. doi: 10.1186/s13020-024-00891-4 (PMC10845799; doi:10.1186/s13020-024-00891-4)
Supplement: Supplementary file 1 — Additional file 1: Table S1. Primers used in the qPCR. Table S2. Primers used in the gene cloning. Table S3. The R2R3-MYB transcription factor characteristics. Figure S1. Expression patterns of anthraquinone biosynthesis pathway in different tissues. A: shikimic acid pathway; B; MEP pathway; C: MVA pathway; D: Polyketide pathway. [file 13020_2024_891_MOESM1_ESM.docx]

**Supplementary Tables and Figures.**

**Table S1** Primers used in the qRT-PCR.

| **Sequencing ID** | **Gene** | **Primer sequence (5′–3′)** |
| --- | --- | --- |
| Isoform0022478 | RpMYB58-F | ATGACGGCAGACGAAAAGGGT |
|  | RpMYB58-R | GTGACGCTTAAGCTCGTCCA |
| Isoform0032325 | RpMYB69-F | GCCCAACAAGAAGCCTCATCA |
|  | RpMYB69-R | CATAGGCCATGTGTGGTGGGA |
| Isoform0035185 | RpMYB73-F | GCCCACCACTCTCCGACTTCTA |
|  | RpMYB73-R | CACTCATCCTCCTCACAATTCCC |
| Isoform0039546 | RpMYB81-F | GAGCAACTCCGTTTTCCCCA |
|  | RpMYB81-R | GATGACCCAGAAGGCGAGAA |
| Isoform0039775 | RpMYB82-F | TGCGCGGAGAATGAGAAAG |
|  | RpMYB82-R | ATCCACCTCAACCTGCAGCT |
| Isoform0049516 | RpMYB100-F | AATTTCAACCCCGGTAGCCC |
|  | RpMYB100-R | ATCCGGGAAGCGAAAGACTC |

**Table S2** Primers used in the gene cloning.

| Project | Primer | Primer sequence (5'→3') |
| --- | --- | --- |
| 1305-*RpMYB81* cloning | 1305-*RpMYB81*-F | GCTCTAGAATGGGAAGACAACCATGTTGT |
|  | 1305-*RpMYB81*-R | CGGGATCCTCAGACATTAGAGTTCAACTCTGTG |
| 1305-*RpMYB98* cloning | 1305-*RpMYB98*-F | ACTAGTATGGGAAGGTCTCCTTGTTGTGAG |
|  | 1305-*RpMYB98*-R | CGGGATCCCTATGGAGATACCTTGGCGGTATG |
| pGBKT7-*RpMYB81* cloning | PGBKT7-*RpMYB81*-F | CGGGATCCATGGGAAGACAACCATGTTGT |
|  | PGBKT7-*RpMYB81*-R | CTGCAGTCAGACATTAGAGTTCAACTCTGTG |
| pGBKT7-*RpMYB98* cloning | PGBKT7-*RpMYB98*-F | GGAATTCATGGGAAGGTCTCCTTGTTGTGAG |
|  | PGBKT7-*RpMYB98*-R | CGGGATCCCTATGGAGATACCTTGGCGGTATG |
| Primer of pCAMBIA1305-GFP vector | pCAMBIA1305-GFP-F | AGCCCAGATCAACTAGTC |
|  | pCAMBIA1305-GFP-R | AACTTGTGGCCGTTCACGTC |
| Primer of PGBKT7 vector | PGBKT7-F | ATACGACTCACTATAGGGCGAG |
|  | PGBKT7-R | GACCCGTTTAGAGGCCCCAAGG |

| **Table S3** The R2R3-MYB transcription factor characteristics. | | | | | | | | | | | | |
| --- | --- | --- | --- | --- | --- | --- | --- | --- | --- | --- | --- | --- |
| Gene symbol | Sequencing ID | Protein length (aa) | Molecular weight (Da) | Isoelectric point | Grand average of hydropathicity | Instability index | Fatty coefficient | Alpha helix | Extended strand | Beta turn | Random coil | subcellular localization |
| RpMYB50 | Isoform0000958 | 930 | 105031.85 | 5.37 | -1.005 | 53.62 | 65.67 | 0.5548 | 0.0376 | 0.0194 | 0.3882 | nucleus |
| RpMYB51 | Isoform0008539 | 459 | 51287.64 | 5.64 | -0.652 | 65.52 | 77.17 | 0.342 | 0.0937 | 0.0436 | 0.5207 | nucleus |
| RpMYB52 | Isoform0011574 | 459 | 51315.7 | 5.64 | -0.647 | 65.52 | 77.58 | 0.342 | 0.0915 | 0.0436 | 0.5229 | nucleus |
| RpMYB53 | Isoform0015461 | 268 | 30373.83 | 9.02 | -0.819 | 42.92 | 59.4 | 0.2687 | 0.1493 | 0.0336 | 0.5485 | nucleus |
| RpMYB54 | Isoform0017483 | 298 | 30373.83 | 9.02 | -0.819 | 42.92 | 59.4 | 0.1946 | 0.0839 | 0.0268 | 0.6946 | nucleus |
| RpMYB55 | Isoform0019031 | 363 | 39502.01 | 5.75 | -0.55 | 47.98 | 72.56 | 0.3113 | 0.0496 | 0.0496 | 0.5895 | nucleus |
| RpMYB56 | Isoform0020515 | 363 | 39488.96 | 5.64 | -0.549 | 48.19 | 72.29 | 0.3196 | 0.0523 | 0.0413 | 0.5868 | nucleus |
| RpMYB57 | Isoform0020820 | 380 | 42545.17 | 8.55 | -0.803 | 65.18 | 60.84 | 0.3053 | 0.0395 | 0.0474 | 0.6079 | nucleus |
| RpMYB58 | Isoform0022478 | 298 | 32006.5 | 7.98 | -0.717 | 49.32 | 59.63 | 0.1879 | 0.0839 | 0.0369 | 0.6913 | nucleus |
| RpMYB59 | Isoform0023844 | 433 | 46578.38 | 5.29 | -0.259 | 47.58 | 75.08 | 0.2979 | 0.0531 | 0.0323 | 0.6166 | nucleus |
| RpMYB60 | Isoform0024319 | 319 | 35128.19 | 6.4 | -0.755 | 48.76 | 60.69 | 0.2884 | 0.0658 | 0.0282 | 0.6176 | nucleus |
| RpMYB61 | Isoform0024803 | 363 | 39487.98 | 5.75 | -0.549 | 47.98 | 72.29 | 0.3003 | 0.0579 | 0.0413 | 0.6006 | nucleus |
| RpMYB62 | Isoform0025852 | 367 | 41660.23 | 5.62 | -0.808 | 49.54 | 63.24 | 0.3052 | 0.1281 | 0.0763 | 0.4905 | nucleus |
| RpMYB63 | Isoform0026539 | 268 | 30359.8 | 9.02 | -0.819 | 43.24 | 59.4 | 0.2836 | 0.1306 | 0.0485 | 0.5373 | nucleus |
| RpMYB64 | Isoform0026993 | 330 | 38512.17 | 9.11 | -0.978 | 65.54 | 69.24 | 0.5879 | 0.0394 | 0.0333 | 0.3394 | nucleus |
| RpMYB65 | Isoform0029107 | 417 | 45130.65 | 5.37 | -0.404 | 53.57 | 73.96 | 0.3285 | 0.0624 | 0.0336 | 0.5755 | nucleus |
| RpMYB66 | Isoform0031201 | 317 | 35543.63 | 5.24 | -0.712 | 49.08 | 67.98 | 0.2618 | 0.1262 | 0.0599 | 0.5521 | nucleus |
| RpMYB67 | Isoform0031832 | 322 | 34839.64 | 8.25 | -0.439 | 53.19 | 74.53 | 0.3075 | 0.0683 | 0.0466 | 0.5776 | nucleus |
| RpMYB68 | Isoform0031958 | 359 | 39453.18 | 6.5 | -0.636 | 54.02 | 68.83 | 0.39 | 0.0529 | 0.0474 | 0.5097 | nucleus |
| RpMYB69 | Isoform0032325 | 298 | 31996.5 | 8.64 | -0.708 | 47.47 | 59.6 | 0.1779 | 0.0839 | 0.0302 | 0.7081 | nucleus |
| RpMYB70 | Isoform0032761 | 284 | 32214.75 | 9.11 | -0.795 | 43.88 | 78.66 | 0.2817 | 0.1021 | 0.0528 | 0.5634 | nucleus |
| RpMYB71 | Isoform0033379 | 365 | 40422.66 | 5.39 | -0.61 | 43 | 74.79 | 0.2329 | 0.1616 | 0.0904 | 0.5151 | nucleus |
| RpMYB72 | Isoform0034953 | 394 | 43148.76 | 6.01 | -0.734 | 42.87 | 62.16 | 0.3223 | 0.099 | 0.0533 | 0.5254 | nucleus |
| RpMYB73 | Isoform0035185 | 391 | 42764.49 | 5.97 | -0.683 | 43.87 | 64.37 | 0.3478 | 0.1023 | 0.0512 | 0.4987 | nucleus |
| RpMYB74 | Isoform0035222 | 315 | 34196.05 | 9.4 | -0.331 | 63.56 | 75.65 | 0.3048 | 0.0603 | 0.0349 | 0.6 | nucleus |
| RpMYB75 | Isoform0035871 | 322 | 34749.51 | 8.25 | -0.451 | 54.42 | 74.57 | 0.2857 | 0.0839 | 0.0311 | 0.5994 | nucleus |
| RpMYB76 | Isoform0035997 | 268 | 29980.93 | 8.86 | -0.656 | 57.13 | 67.76 | 0.25 | 0.0485 | 0.0597 | 0.6418 | nucleus |
| RpMYB77 | Isoform0036631 | 303 | 32594.65 | 8.65 | -0.567 | 54.71 | 67.59 | 0.2739 | 0.0726 | 0.033 | 0.6205 | nucleus |
| RpMYB78 | Isoform0036788 | 332 | 36780.91 | 6.98 | -0.653 | 61.88 | 60.33 | 0.2078 | 0.1265 | 0.0542 | 0.6114 | nucleus |
| RpMYB79 | Isoform0039216 | 268 | 29492.06 | 7.62 | -0.676 | 57.51 | 70.04 | 0.2799 | 0.0634 | 0.0634 | 0.5933 | nucleus |
| RpMYB80 | Isoform0039268 | 295 | 31714.69 | 8.81 | -0.547 | 60.75 | 65.76 | 0.2847 | 0.0678 | 0.0339 | 0.6136 | nucleus |
| RpMYB81 | Isoform0039546 | 316 | 35444.5 | 5.24 | -0.727 | 49.21 | 67.28 | 0.2722 | 0.1044 | 0.057 | 0.5665 | nucleus |
| RpMYB82 | Isoform0039775 | 248 | 27291.05 | 8.71 | -0.631 | 52 | 77.82 | 0.2661 | 0.1371 | 0.0605 | 0.5363 | nucleus |
| RpMYB83 | Isoform0040022 | 320 | 34549.32 | 8.58 | -0.449 | 54.97 | 74.41 | 0.2812 | 0.0719 | 0.0469 | 0.6 | nucleus |
| RpMYB84 | Isoform0041071 | 246 | 27370.04 | 8.09 | -0.636 | 37.71 | 80.08 | 0.2683 | 0.1138 | 0.0691 | 0.5488 | nucleus |
| RpMYB85 | Isoform0041164 | 302 | 32423.45 | 8.87 | -0.573 | 54.22 | 66.85 | 0.2748 | 0.0695 | 0.0364 | 0.6192 | nucleus |
| RpMYB86 | Isoform0041378 | 321 | 34708.55 | 8.58 | -0.421 | 53.32 | 75.08 | 0.2741 | 0.0872 | 0.0312 | 0.6075 | nucleus |
| RpMYB87 | Isoform0041734 | 317 | 36359.61 | 6.8 | -0.816 | 38.84 | 68.3 | 0.2492 | 0.1041 | 0.0852 | 0.5615 | nucleus |
| RpMYB88 | Isoform0041950 | 314 | 34053.84 | 9.26 | -0.319 | 62.9 | 75.89 | 0.293 | 0.0764 | 0.0478 | 0.5828 | nucleus |
| RpMYB89 | Isoform0042058 | 268 | 30058.68 | 8.15 | -0.794 | 42.91 | 67.01 | 0.3321 | 0.0448 | 0.0858 | 0.5373 | nucleus |
| RpMYB90 | Isoform0042092 | 272 | 30403.56 | 9.08 | -0.599 | 48.89 | 82.9 | 0.261 | 0.1544 | 0.0699 | 0.5147 | nucleus |
| RpMYB91 | Isoform0043078 | 302 | 32465.53 | 8.87 | -0.557 | 54.86 | 67.81 | 0.2848 | 0.0662 | 0.0298 | 0.6192 | nucleus |
| RpMYB92 | Isoform0044197 | 315 | 34085.81 | 8.58 | -0.453 | 55.02 | 76.19 | 0.2698 | 0.0762 | 0.0381 | 0.6159 | nucleus |
| RpMYB93 | Isoform0044746 | 294 | 32876.44 | 8.08 | -0.603 | 51.29 | 73.71 | 0.3265 | 0.0544 | 0.051 | 0.568 | nucleus |
| RpMYB94 | Isoform0046442 | 278 | 31063.79 | 6.9 | -0.715 | 50.96 | 68.42 | 0.3741 | 0.054 | 0.036 | 0.536 | nucleus |
| RpMYB95 | Isoform0046603 | 269 | 30171.84 | 8.15 | -0.777 | 42.79 | 68.22 | 0.3011 | 0.0595 | 0.0632 | 0.5762 | nucleus |
| RpMYB96 | Isoform0046748 | 237 | 26754.1 | 7.06 | -0.67 | 49.1 | 76.67 | 0.3376 | 0.0422 | 0.0802 | 0.5401 | nucleus |
| RpMYB97 | Isoform0047916 | 247 | 26985.58 | 6.31 | -0.497 | 53.78 | 76.8 | 0.3563 | 0.1093 | 0.0688 | 0.4656 | nucleus |
| RpMYB98 | Isoform0049173 | 280 | 30505.55 | 8.68 | -0.417 | 58.02 | 76.71 | 0.2 | 0.0929 | 0.0929 | 0.6143 | nucleus |
| RpMYB99 | Isoform0049272 | 238 | 27639.67 | 7.65 | -1.237 | 66.48 | 59.45 | 0.4286 | 0.063 | 0.0714 | 0.437 | nucleus |
| RpMYB100 | Isoform0049516 | 303 | 32552.57 | 8.65 | -0.582 | 54.07 | 66.63 | 0.2871 | 0.0462 | 0.0297 | 0.637 | nucleus |
| RpMYB101 | Isoform0052109 | 237 | 26740.03 | 6.67 | -0.668 | 48.08 | 76.67 | 0.3544 | 0.0591 | 0.0802 | 0.5063 | nucleus |


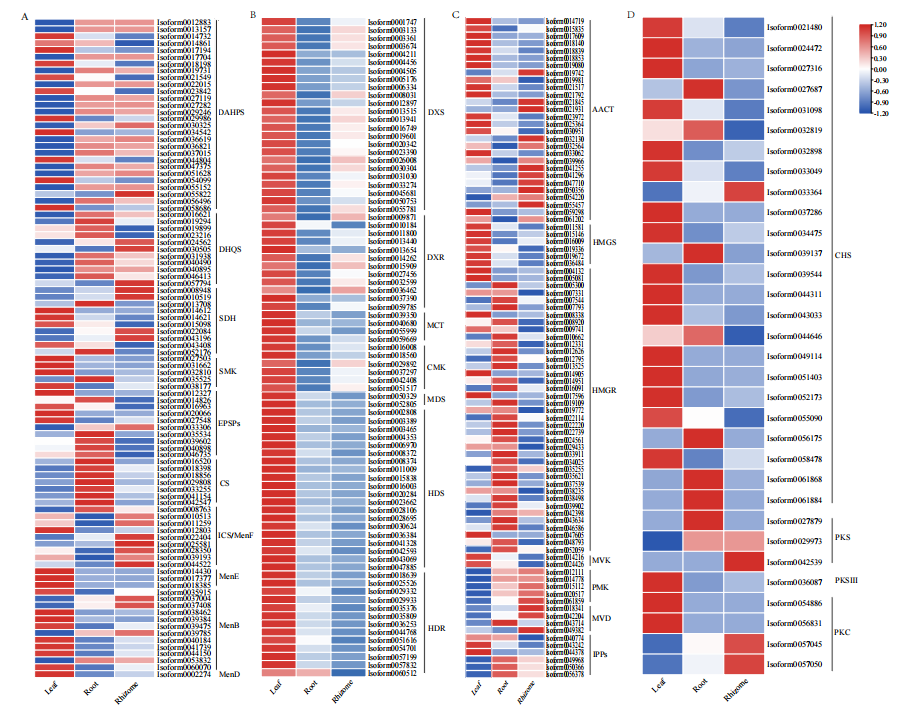


**Figure S1**. Expression patterns of anthraquinone biosynthesis pathway in different tissues. A: shikimic acid pathway; B; MEP pathway; C: MVA pathway; D: Polyketide pathway.
